# Supplementary figures and images for: Epigenetics is all you need: A transformer to decode chromatin structural compartments from the epigenome
Source: PLoS Comput Biol. 2025 Dec 3;21(12):e1012326. doi: 10.1371/journal.pcbi.1012326 (PMC12685209; doi:10.1371/journal.pcbi.1012326)

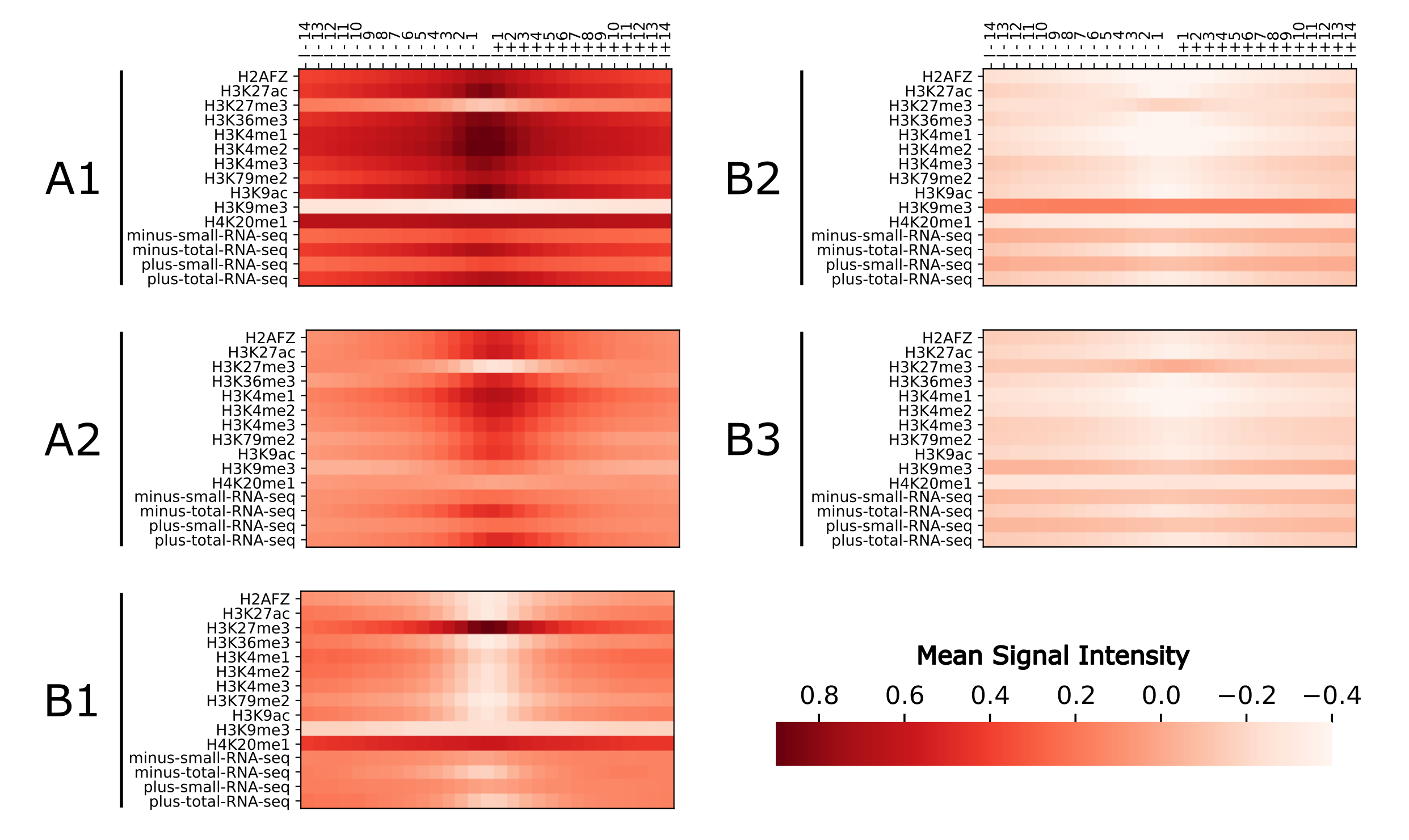

Supplement: S1 Fig — (TIFF) [file pcbi.1012326.s001.tif]

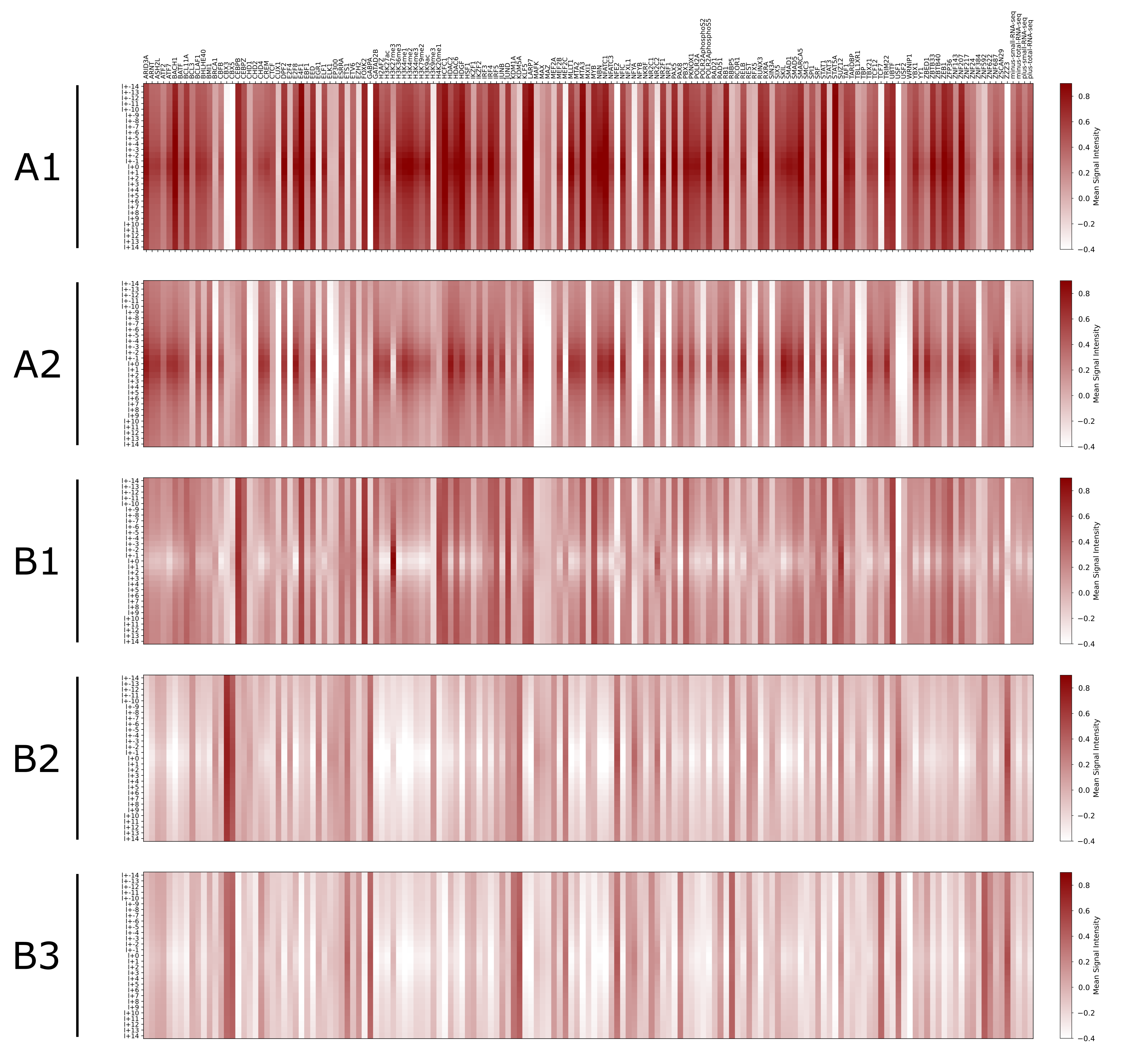

Supplement: S2 Fig — (TIFF) [file pcbi.1012326.s002.tif]

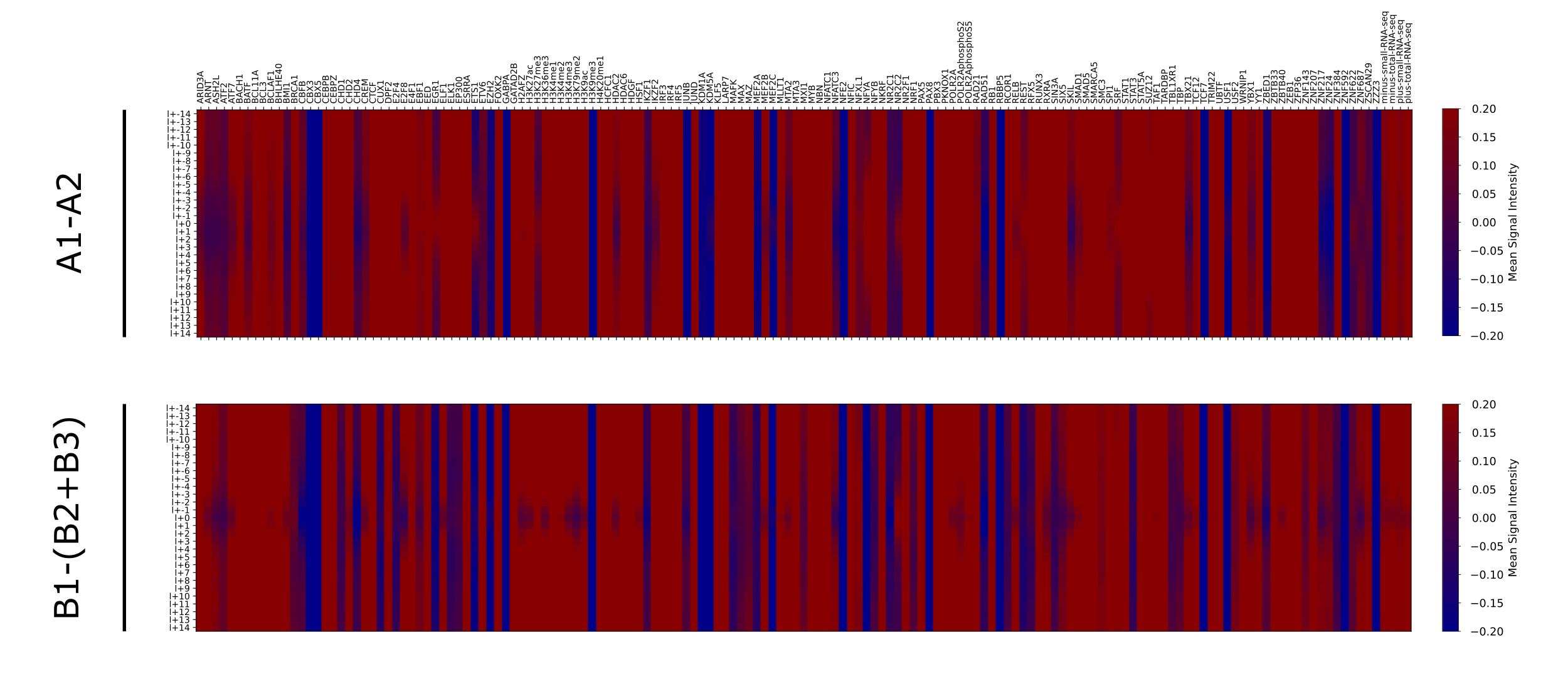

Supplement: S3 Fig — (TIFF) [file pcbi.1012326.s003.tif]

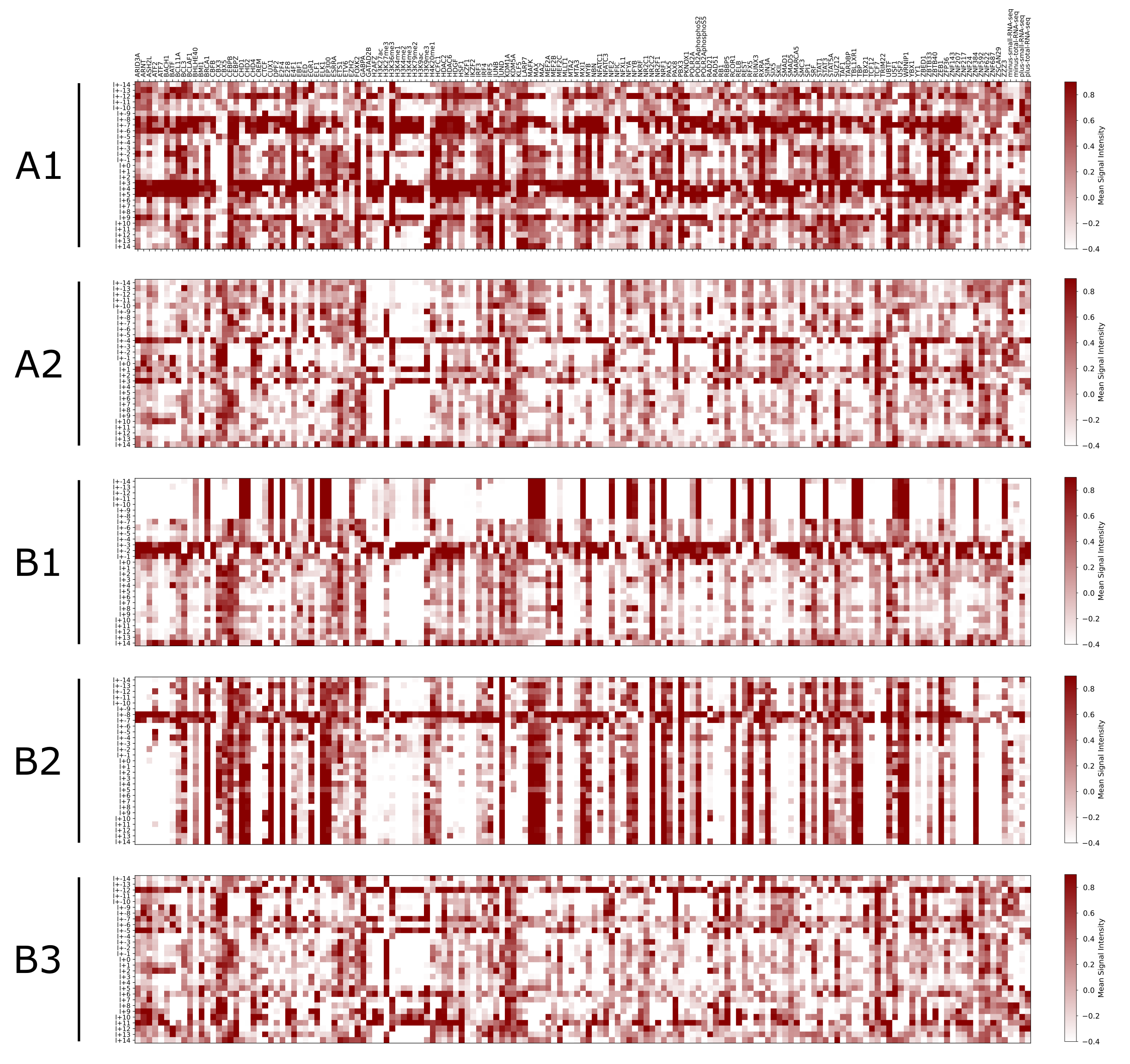

Supplement: S4 Fig — (TIFF) [file pcbi.1012326.s004.tif]

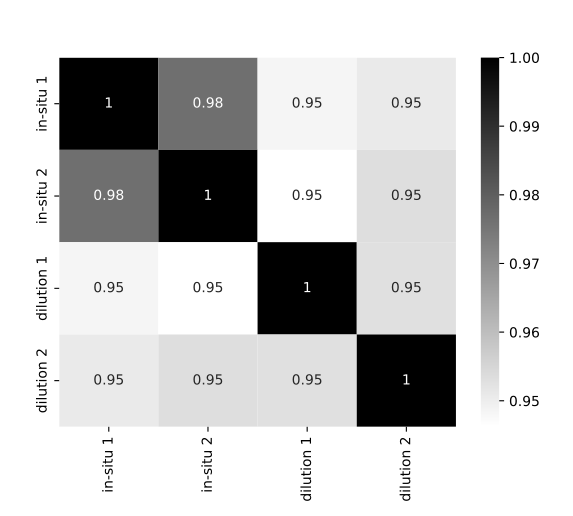

Supplement: S5 Fig — (TIFF) [file pcbi.1012326.s005.tif]

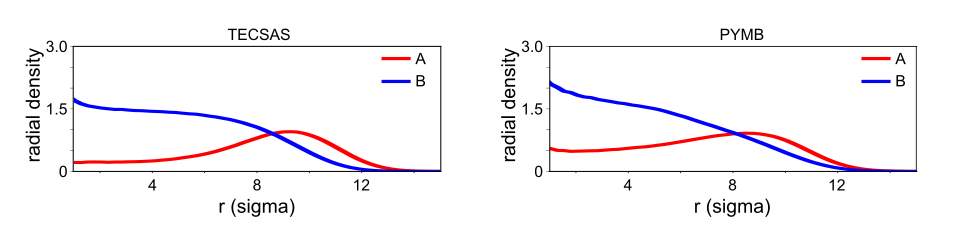

Supplement: S6 Fig — (TIFF) [file pcbi.1012326.s006.tif]

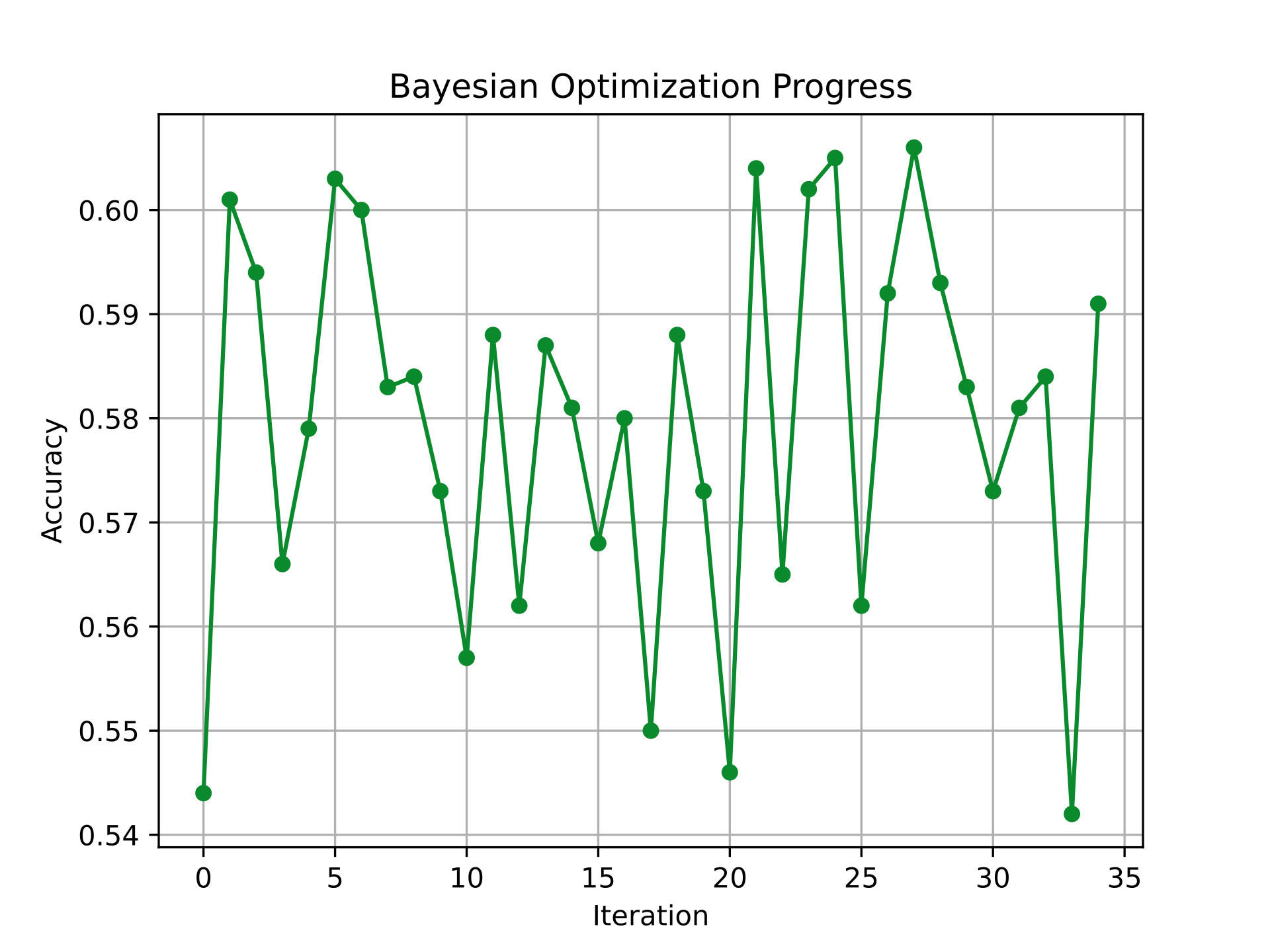

Supplement: S7 Fig — (TIFF) [file pcbi.1012326.s007.tif]

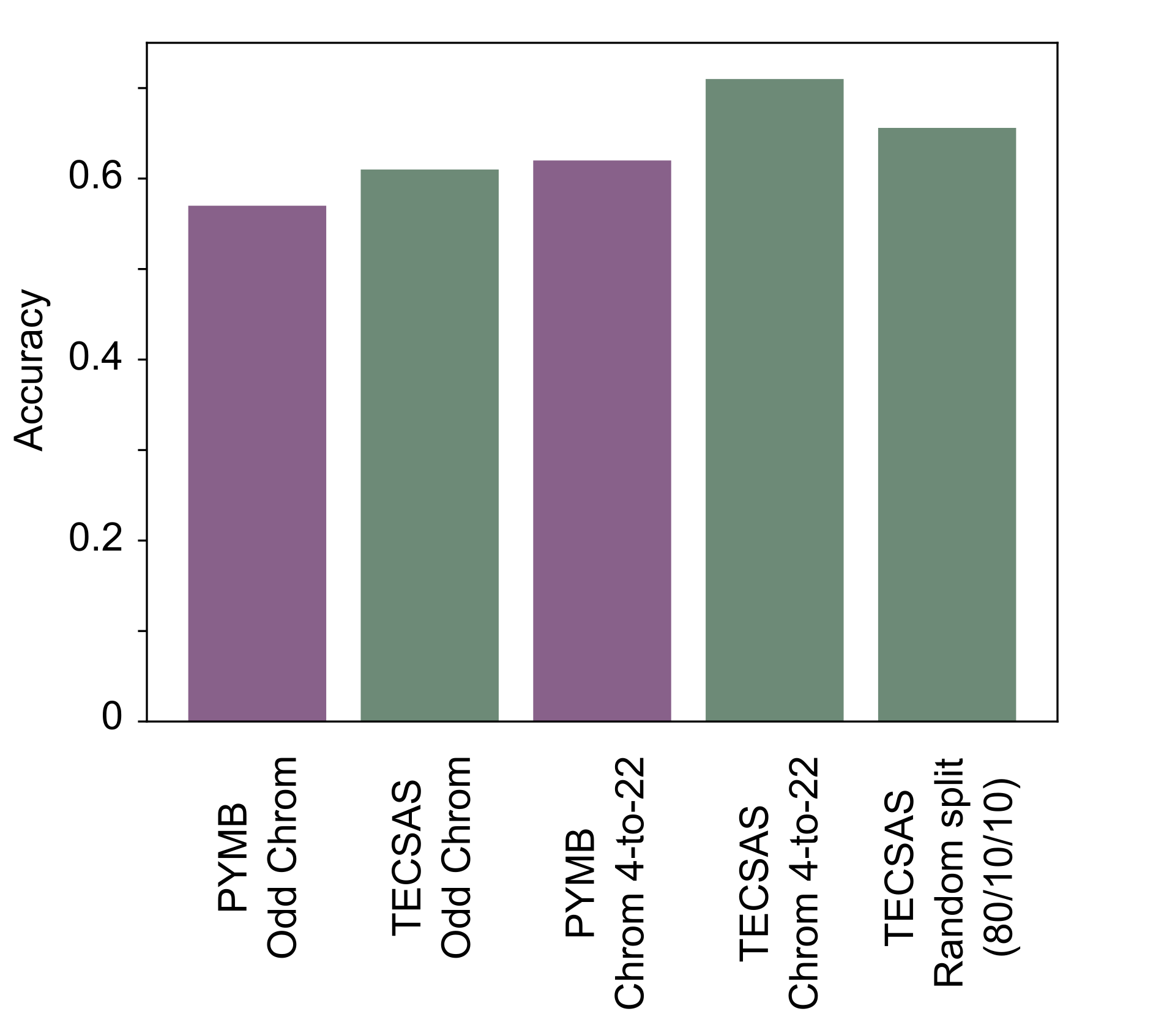

Supplement: S8 Fig — (TIFF) [file pcbi.1012326.s008.tif]

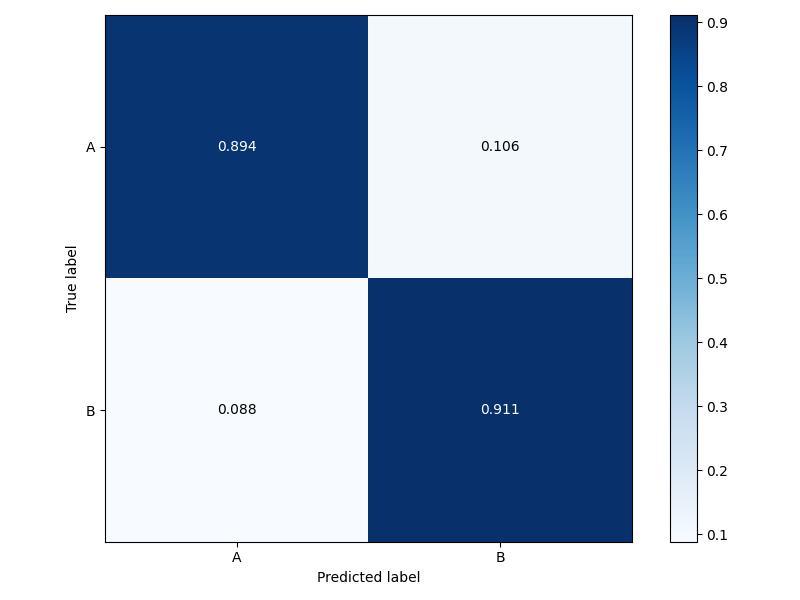

Supplement: S9 Fig — (TIFF) [file pcbi.1012326.s009.tif]

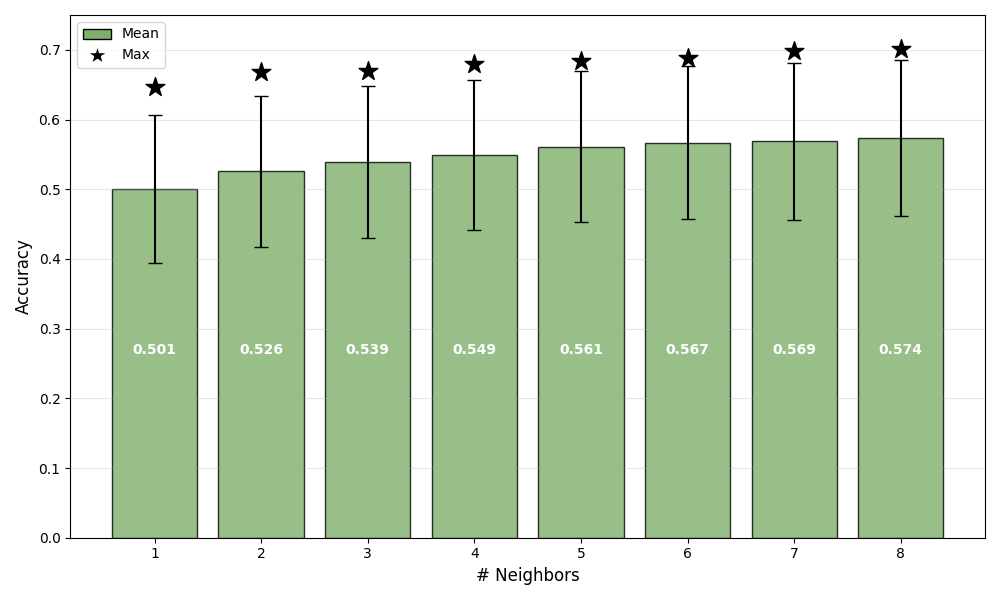

Supplement: S10 Fig — (TIFF) [file pcbi.1012326.s010.tif]

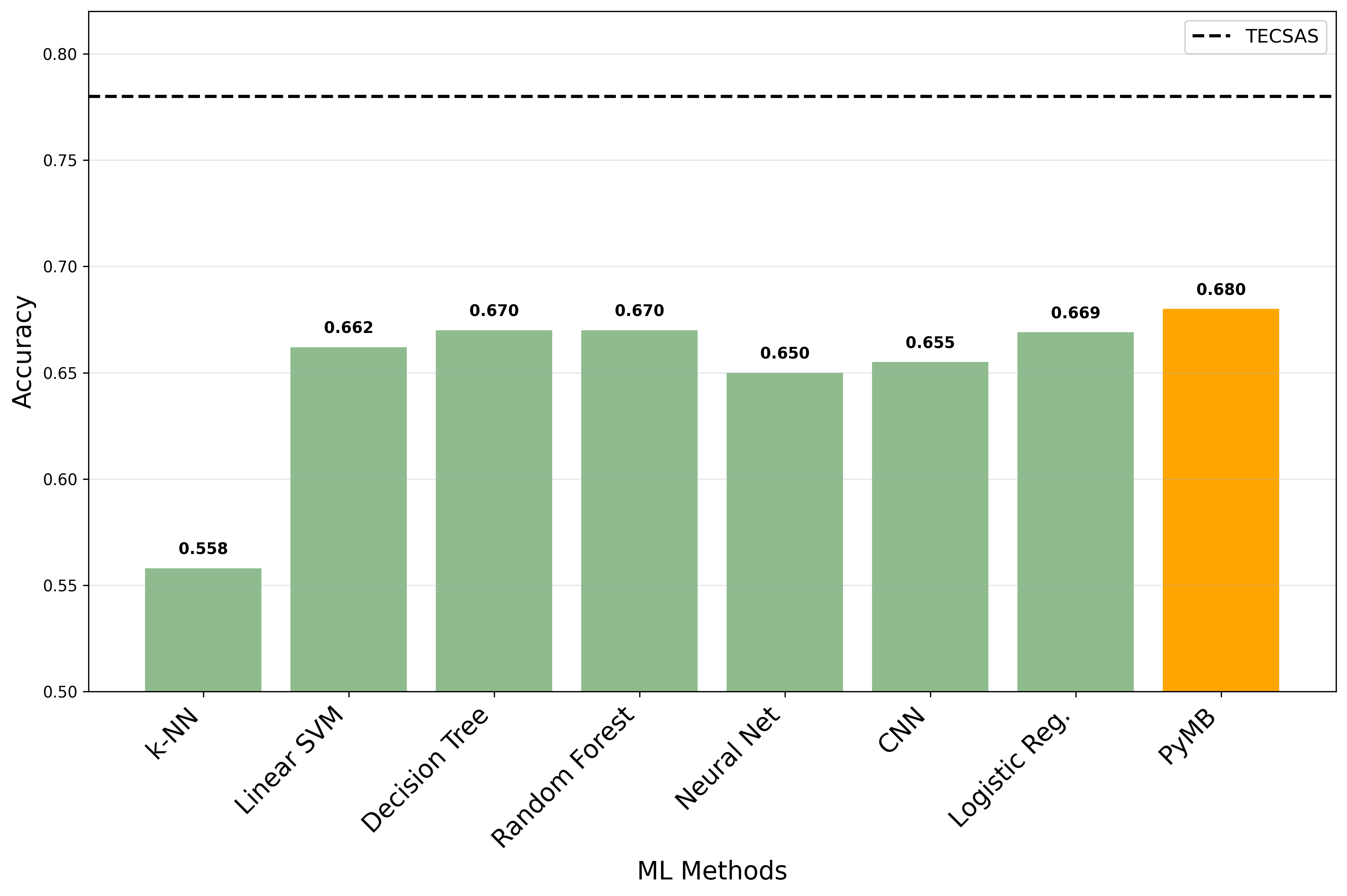

Supplement: S11 Fig — (TIFF) [file pcbi.1012326.s011.tif]

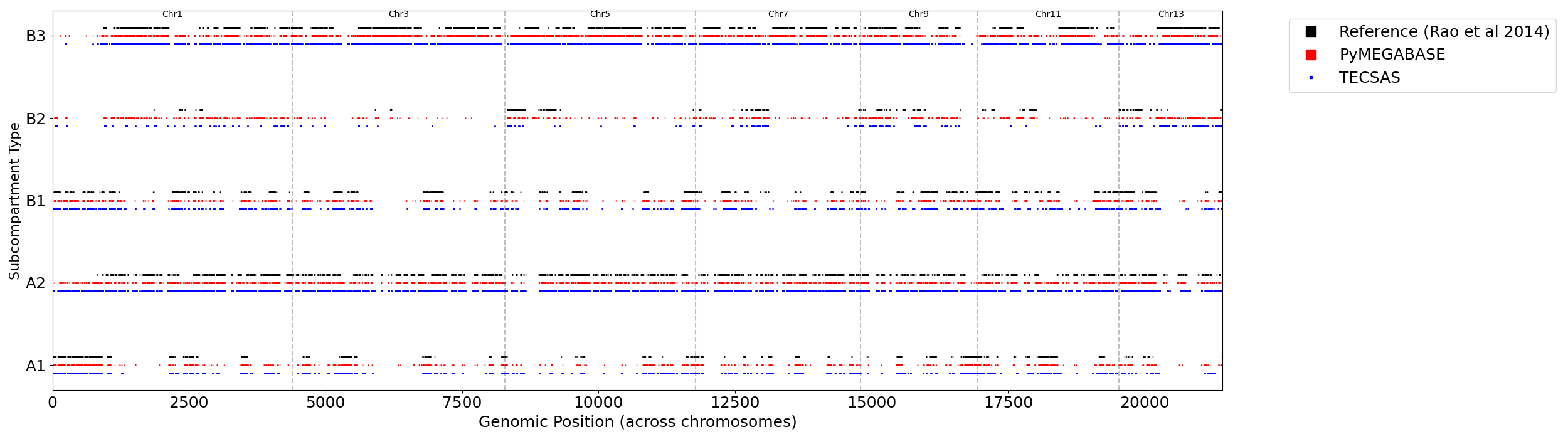

Supplement: S12 Fig — (TIFF) [file pcbi.1012326.s012.tif]

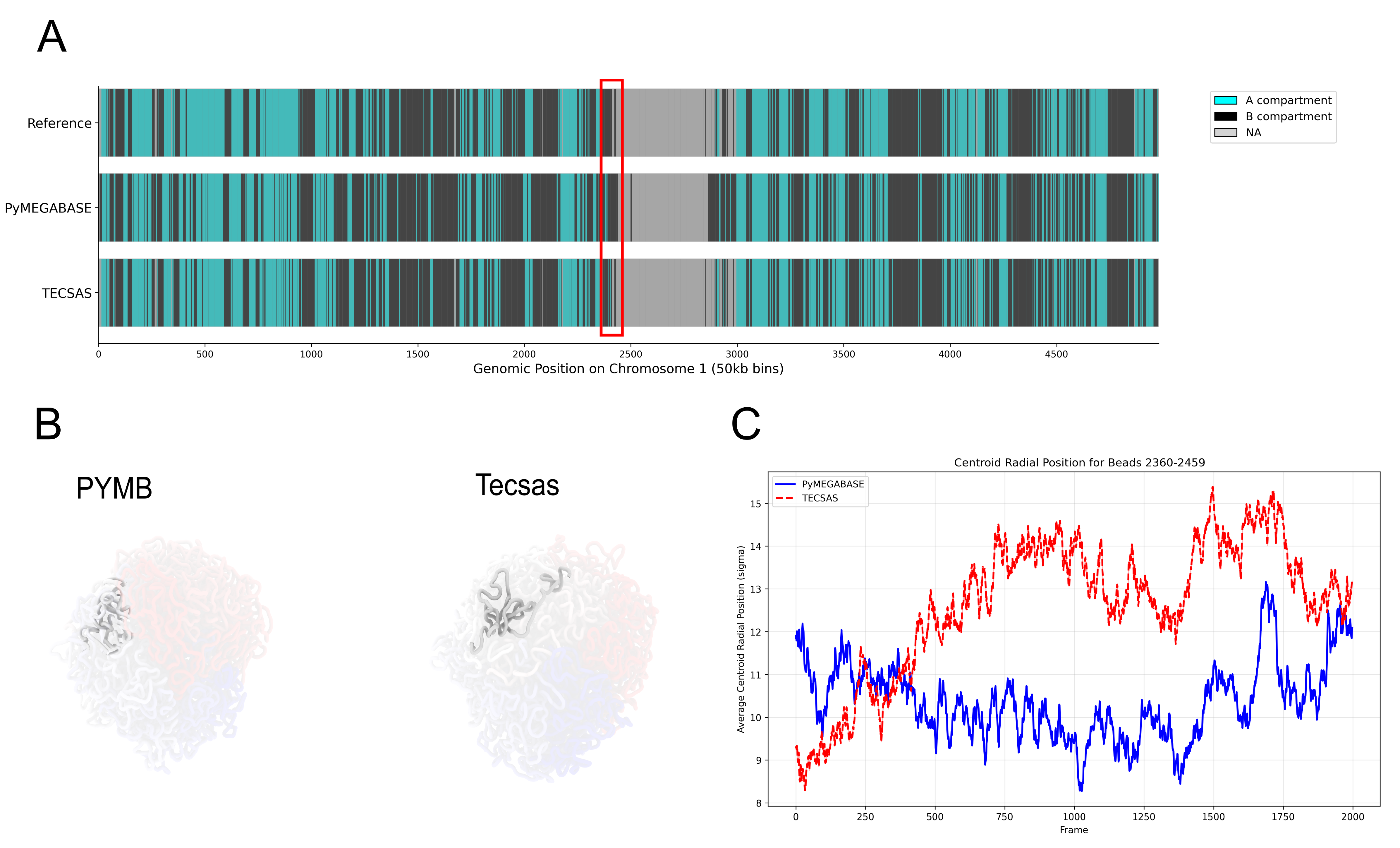

Supplement: S13 Fig — (TIFF) [file pcbi.1012326.s013.tif]

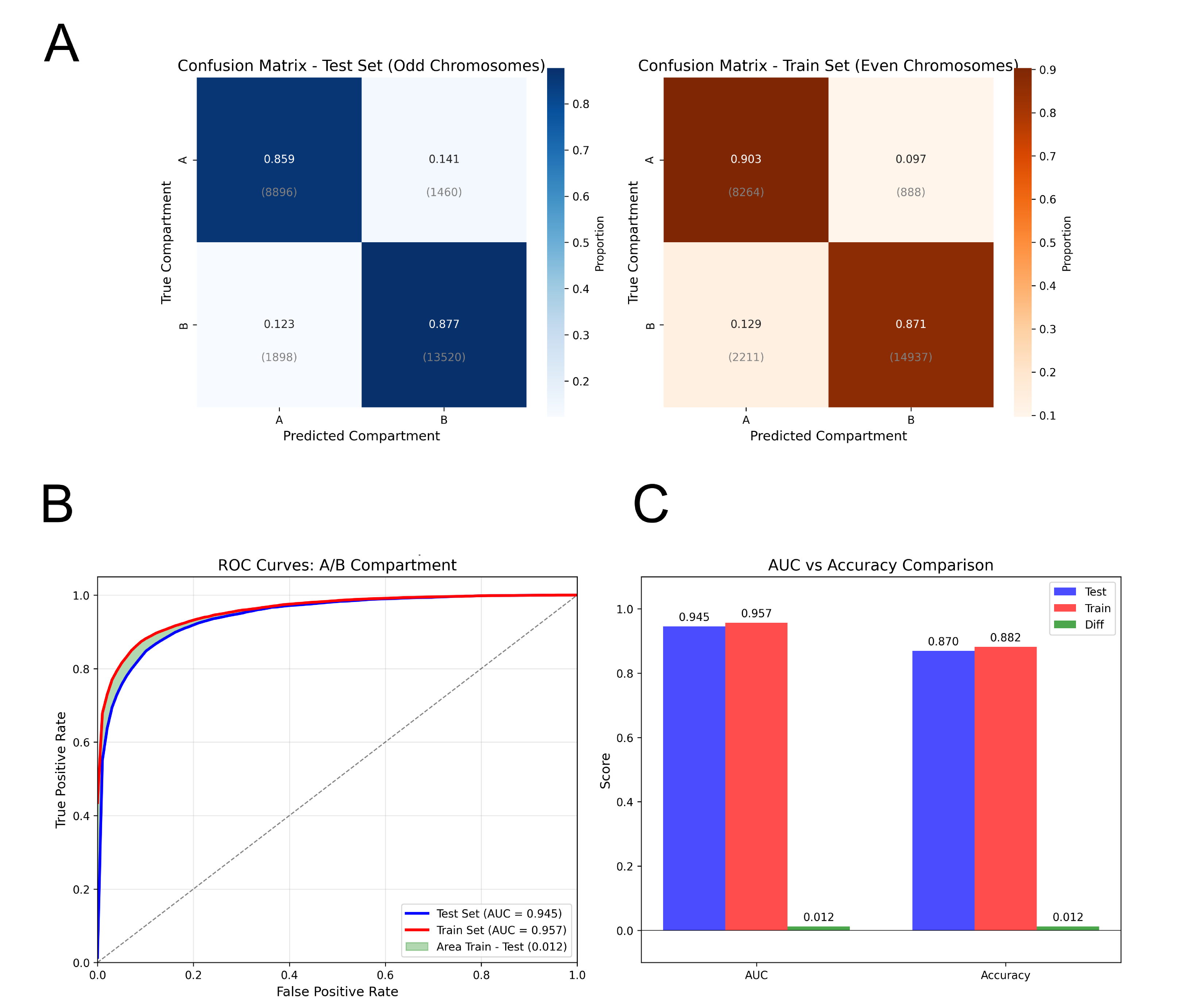

Supplement: S14 Fig — (TIFF) [file pcbi.1012326.s014.tif]
