# Supplementary material for: Epigenetics is all you need: A transformer to decode chromatin structural compartments from the epigenome
Source: PLoS Comput Biol. 2025 Dec 3;21(12):e1012326. doi: 10.1371/journal.pcbi.1012326 (PMC12685209; doi:10.1371/journal.pcbi.1012326)
Supplement: S1 Table — (PDF) [file pcbi.1012326.s015.pdf]

S1 Table. Hyperparameter accuracy comparison using Bayesian Optimization.

Iteration	Accuracy	Trans. layers	F.F. Dim.	# heads	Emb. Dim	lr	dropout
Empirical	0.61	2	64	8	128	2.5	0.01
1	0.544	3	24	2	12	0.6077	0.8681
2	0.601	3	120	2	18	1.835	0.323
3	0.594	3	56	4	24	0.9001	0.433
4	0.566	2	82	2	14	0.1487	0.2527
5	0.579	2	54	6	12	0.9097	0.7364
6	0.603	3	126	2	24	0.789	0.03329
7	0.6	1	114	6	90	1.577	0.04214
8	0.583	1	126	6	12	1.698	0.4483
9	0.584	3	56	2	30	0.4765	0.306
10	0.573	3	104	2	30	0.7702	0.4007
11	0.557	1	122	6	72	0.1007	0.382
12	0.588	2	58	4	8	0.5022	0.09446
13	0.562	1	38	6	90	1.479	0.8044
14	0.587	1	98	6	78	0.1926	0.4081
15	0.581	2	110	2	24	1.439	0.2504
16	0.568	3	114	4	16	1.053	0.6968
17	0.58	3	56	2	30	0.515	0.3136
18	0.55	3	126	2	26	1.824	0.7959
19	0.588	3	118	2	16	1.678	0.3414
20	0.573	1	114	6	90	1.963	0.1901
21	0.546	3	126	2	26	0.2074	0.5626
22	0.604	2	98	6	84	0.5084	0.2646
23	0.565	3	96	2	6	1.364	0.3025
24	0.602	2	104	4	36	0.7807	0.5439
25	0.605	1	34	4	52	0.3557	0.6503
26	0.562	2	56	6	24	1.08	0.5236
27	0.592	2	98	6	78	0.6513	0.08031
28	0.606	3	24	6	84	0.6283	0.3007
29	0.593	2	104	4	36	0.6245	0.6283
30	0.583	1	98	6	84	0.502	0.5674
31	0.573	2	48	2	16	0.8638	0.577
32	0.581	3	24	6	84	0.9185	0.6461
33	0.584	3	24	6	84	0.7757	0.5703
34	0.542	2	34	6	36	1.199	0.7958
35	0.591	2	104	6	54	0.4303	0.2689
