# Supplementary material for: Epigenetics is all you need: A transformer to decode chromatin structural compartments from the epigenome
Source: PLoS Comput Biol. 2025 Dec 3;21(12):e1012326. doi: 10.1371/journal.pcbi.1012326 (PMC12685209; doi:10.1371/journal.pcbi.1012326)
Supplement: S2 Table — (PDF) [file pcbi.1012326.s016.pdf]

S2 Table. Subcompartment distribution for K562 and GM12878.

Subcompartment	# loci	Resolution
GM12878		
A1	7984	50 kbp
A2	11628	50 kbp
B1	6988	50 kbp
B2	8714	50 kbp
B3	17110	50 kbp
K562		
T1	32828	25 kbp
T2	27822	25 kbp
T3	25222	25 kbp
T4	21193	25 kbp
