# Supplementary material for: Epigenetics is all you need: A transformer to decode chromatin structural compartments from the epigenome
Source: PLoS Comput Biol. 2025 Dec 3;21(12):e1012326. doi: 10.1371/journal.pcbi.1012326 (PMC12685209; doi:10.1371/journal.pcbi.1012326)
Supplement: Attachment — Submitted filename: response-TECSAS.pdf [file pcbi.1012326.s017.pdf]

PLoS Comp Bio Editors and reviewers,

We would like to thank the reviewers for their suggestions and comments on our manuscript. Their valuable feedback and constructive recommendations have contributed significantly to improving the clarity of our work. The changes made in the manuscript are marked in red color. We also expanded the Supporting Information by including new figures and details of the analysis and simulation algorithms. In addition, we also included a detailed tutorial for running TECSAS and facilitating its usage for the community. In the following, we present our responses to all of the reviewers' comments.

REVIEWER COMMENTS

REVIEWER 1

Comment: *"Benchmarking to more methods is needed: I realize that the authors have previously published on the same topic, and maybe their previous papers do include more benchmarking, but in the context of this deep-learning paper, I would like to see a lot more benchmarking against much simpler models. I am demanding this following a preprint (see https://www.biorxiv.org/content/10.1101/2024.09.16.613342v3) which shows that e.g. fancy deep-learning foundation models do yet not outperform simple linear regression on specific tasks. So, how about if the authors were to use the input data in exactly the same form as used for TECSAS, but instead of feeding it through a transformer architecture, why not use something simpler like Random Forests, a simple MLP or penalized linear regression? Doing so would give the reader a much better understanding of how much 'deep-learning' per-se can improve upon 'non deep-learning' methods. So, I would like to see more extensive benchmarking against these other ML-methods."*

Author reply: Thank you for your comment. As the reviewer correctly pointed out, our group has previously published on the topic, and benchmarks were generated and compared with other models. For the PyMEGABASE (PYMB) tool that employs a Potts model energy function, we benchmark PYMB with other simpler architectures using the same input data and format. PYMB scored the best among all tested algorithms for predicting compartments and subcompartments based only on epigenetic tracks. Here, our initial idea was to expand the capability of these predictions using a more elaborated network, and this is the reason we initially compared it only with PYMB, which was the best available tool for that task until TECSAS. To address the reviewer's comment, we modified the main paper to mention that other simple models have been tested. The following comment was included in the section 2.2:

Previous benchmarks have shown that PYMB outperforms less complex ma-

chine learning techniques such as Random Forest or linear regression [23]. In this regard, PYMB is used as a default model for predicting compartments and subcompartments using only 1D tracks. We used the PYMB benchmark as our baseline to test TECSAS's capability to better understand the nature of epigenetic data using more complex neural network architecture.
First paragraph, section 2.2

Comment: *"Type of benchmarking has limitations: Related to the previous point, I am always uneasy when authors split the set of genomic loci up into training and test sets, when the test sets derive from the same genome. Similar to the preprint above, there have been similar articles published indicating that this form of "validation" can be very biased, i.e. the predictive accuracies are driven by specific features relating to the given cell-type, which do not generalize to other cell-types. Indeed, I notice that in the first analyses in this MS, the authors do not cross-validate a model trained on one cell-type, on another. Rather, it seems that the authors retrain the model for each cell-type separately. Apologies if I misunderstood, but additional clarification to this effect would be really important."*

Author reply: As the reviewer correctly points out, there are studies that show how certain epigenetic marks behave differently in different cell lines. Indeed, this is a limitation of our approach. However, our previous paper on PYMB and this work also shows that the epigenetic marks learned in GM12878 cell line are transferable to other cell lines. This can also be understood in terms of the amount of data used in the training and validation sets. As presented in our results, it is not necessary to include every epigenetic track to predict chromatin compartments A and B accurately. Instead, a subset of tracks can already provide enough information for the model to assign the correct compartment with a higher probability. Furthermore, in the case of XADS, we trained the model on data from H1, K562, and HCT116 (reducing the cell bias in the training set) and were tested in IMR-90 (fully independent from the training set).

Comment: *"Higher order interactions vs pairwise: Related to the previous point, I understand that the Potts Model (PYMB) would only capture pairwise interactions between epigenetic signals right, and that the deep-learning method could capture higher-order interactions that are important for the improvement in prediction. However, the need for higher-order interactions (ie beyond pairwise) for the improved prediction is not clearly borne out from the main figures, in particular Fig.3D-E. Fig.3E only shows the importance of considering large neighborhoods, whilst close inspection of Fig.3D suggests that it is the pairwise interaction of H3K27me3 and H3K9me3 that drives the prediction of this particular locus. In other words, I do apologize if I missed it, but from*

the main figures I can't glean a higher-order interaction driving predictive performance, which hence would suggest that maybe the improvement over PYMB has more to do with the use of larger neighborhoods?"

Author reply: Thank you for your comment. We understand the confusion - as higher order interactions is interpreted differently in the ML model. As the reviewer correctly points out, the Potts model only has pairwise interactions, for example, the subcompartment node with H3K4me3 signal at locus L-1 node have a energy cost for interacting. In the case of TECSAS, there is higher order interactions if we think about the network as a set of nodes interconnected nodes (although the connections are highly non-linear). For instance, the connection between the H3K4me3 signal at locus L-1 node and RNASeq signal at locus L+4 are interacting based on the attention matrix, moreover that connection is carried over towards the prediction of the subcomparment, then creating a highly non-linear interaction between the two data nodes and the subcompartment node. To clarify this point in the article, we introduced the following into the main text:

> **This allows the model to learn complex relationships between multiple epigenetic features across different genomic loci. Moreover, the attention map creates a way for the model to connect, in a highly non-linear manner, sets of three nodes: two data nodes and the output subcompartment node - this process bypasses the limitation of PYMB pairwise linear interactions.**
> Third paragraph, section 2.2

Comment: *"Fig.3B: some clarification as to which particular experiments were removed and how exactly would help clarify this, specially in the main text where it is not clearly specified. So, for instance, were specific histone marks removed or what exactly is meant by "experiment"?"*

Author reply: Thank you for pointing out this confusing part of the manuscript. By 'experiment' we mean any ChIP-Seq or RNA-Seq assay track. For the panel 3B, we wanted to showcase the performance of the model when only certain set of experiments are available, no other cell type will have the same set of Chip-Seq and RNA-Seq tracks available. To achieve this, we first randomly selected 20 different sets of 2, ..., 20 of experiments from GM12878 available list (156 in total). For each set, we trained the model and calculated the accuracy of the model at predicting the subcompartments in the test split (following the same train/test/validation split as previous results 80/10/10). To clarify this experiment, we introduced the following into the main text:

> **Further, we compare to PYMB the accuracy of TECSAS with different number of experimental tracks, including Histone Modification Chip-Seq, Transcription Factor Chip-Seq and RNA-Seq, here we refer as "experiment" to each of these**

tracks. To do so, we randomly selected 20 sets of 2, ..., 20 experiments and trained the TECSAS, then computing the accuracy on the test set. Figure 3B shows that regardless of the number of experiments used TECSAS outperforms PYMB. This suggests that the Transformer architecture employed by TECSAS contributes to improved prediction accuracy even when using a limited epigenomic context. Moreover, this benchmark is of particular interest as most of the cell types in the ENCODE portal have available only a small subset of the experiments found for GM12878.

First paragraph, section 2.2

Comment: *"Fig.4: please check labels as I think they are wrong. "*

Author reply: Thank you for pointing this out. We fixed the caption of the figure to accurately refer to each panel. Now it reads:

(A) Compartment annotations from TECSAS, PYMB and experimental Hi-C around the chr4:36-37Mbp segment. (B) Representative structure of chromosome 4 from simulations based on TECSAS and PYMB predictions, highlighting the positioning of the chr4:36-37Mbp segment. (C) Distribution of radial positioning of the chr4:36-37Mbp segment on the simulated ensemble based on TECSAS and PYMB annotations.

Caption, Figure 4.

Comment: *"Section 2.3: In this section, the authors state that "...on GM12878 data, while the last linear layer was trained on combined data from K562, H1, and HCT116 cells. The high accuracy achieved in predicting associated domains suggests that the relationships between epigenomic features and structural annotations learned by TECSAS in GM12878 are transferable to other cell types." I am not sure this is correct. If you want to show that the structural annotations are transferable to other cell-types, please do NOT use the 2 other cell-types (say H1 and HCT116) in the training process. It would be more convincing if you train on one cell-type only and then demonstrate predictive ability in the other two. See point-2 above."*

Author reply: We apologize for the confusing phrasing. For the prediction of XADs, we are only training the last layer of the model, while keeping the transformer encoder parameters fixed. The transferability we refer here is of the epigenetic interpretation performed by the encoder layer - which seems to be transferable to other type of predictions (not only subcompartments) and in other cell lines (K562, H1, and HCT116). Moreover, we further show the transferability of the

model by predicting in IMR-90 (which is fully separated from the training set), where the XADs are predicted and validated based on the relative positioning to their respective nuclear bodies. To clarify the transferability statements, we added the following modifications to the text:

> **The high accuracy achieved in predicting associated domains suggests that the interpretation of epigenomic features learned by the encoder in GM12878 are transferable to other cell types and for the prediction of other structural annotations**
> Second paragraph, section 2.3

Comment: *"Methods need more clarity: I would like to see more details in the Methods section on how the 10% validation set was used. I presume that the 10% test set is the only truly blinded set, and that the 10% validation set was used to optimize parameters, but which parameters these were is unclear."*

Author reply: As the reviewer points out, the validation set was used to select the parameters of the model after training. That is, the resulting parameters of the model are defined as those whose validation loss is the lowest. As expected, the test set is not used during training. We expanded the methodology correspondingly.

> **For the figures in the main text, the genome is split randomly forming training, validation, testing sets corresponding to 80%, 10% and 10% of all the loci, respectively. The validation set is used to choose the parameters of the model (corresponding to the lowest validation loss during training) and the test set is not used during training. Figures in the SI explore different data splits.**
> First paragraph, Machine learning Implementation - Methodology

Comment: *"Some justification for model parameters would be good: In Methods the authors state "TECSAS employs a linear layer for the token embedding process, the embedding dimension is set to 128. This is then followed by a transformer encoder block made of two transformer encoder layers with 8 head each. A linear layer reduces the transformer encoder output to the subcompartment output layers. The dimension of the feedforward layer in the transformer encoder is 64." Could the authors please explain the rationale for these numbers: 128, two transformer encoder layers with 8 heads each, 64. Can they be varied and if so, how does it affect performance? What features of the input data justify these specific numbers?"*

Author reply: Thank you for pointing this out - the current hyperparameters were initially chosen empirically based on multiple rounds of training. To explore the hyperparameter space more fairly, we implemented Bayesian optimization to explore and exploit set of hyperparameters that can turn beneficial for higher accuracy. We included an SI Figure (Figure S7) showing that the model is sensitive to the selection of hyperparameters, yet it is able to maintain predictive power regardless of the selection (within the tested margins). Importantly, the hyperparameters set used in the paper were not sampled during the Bayesian optimization, but they achieve similar degree of accuracy as the top selection of the optimization process - highlighting that the current selection is a good set of hyperparameters.

REVIEWER 2

Comment: *"The robustness of the model needs clearer validation. The manuscript shows good performance on GM12878 and K562, but should include at least one additional cell type for validation to demonstrate broader applicability."*

Author reply: Thank you for your comment. As the manuscript shows, the encoder model was used in more than GM12878 and K562, which includes H1 (NADs and LADs), HCT116 (NADs and LADs) and IMR-90 (NADs, LADs, SPADs). These results highlight that the prediction of structural annotations can be predicted for multiple cells using this architecture.

Comment: *"The authors should better explain how they handle missing data or noise in the epigenomic signals, as this is a common challenge with experimental data. A sensitivity analysis would strengthen the manuscript."*

Author reply: Given that the resolution of Chip-Seq and RNA-seq experiments usually spans hundreds of base-pairs, the signal at 25 kbp and 50 kbp is reasonable to allow us to predict the (sub)compartments. To address the noise, when available, we averaged out different experimental replicates, and we filtered out extreme values by using the 5th and 95th percentile of the signal as a baseline to reduce outliers.
We would like to address the sensitivity concern using the data from the paper. First, most of the time, the model is highly confident in predicting each subcompartment, as shown in Figure 2C. Moreover, TECSAS fails most often in transition regions, as shown in Figure 2E-F. Together, this implies that the model robust at predicting under normal noise affecting all the genome, yet it's main source of uncertainty comes from a characteristic epigenetic phenomena, i.e. transition region.

Comment: *"The comparison with other recent transformer-based methods like MethylGPT and CpGPT should be expanded slightly to position this work in the current landscape."*

Author reply: Thank you for bringing these models to our attention. We now mention them in the introduction to better portray TECSAS in the deep learning landscape.

> **Moreover, recent work has shown great success in predicting phenotypic features from DNA-sequence (CpGPT [1]) and methylation profiles (MethylGPT [2]) - highlighting the promise of deep-learning architectures, such as Evo models [3, 4], to predict highly complex trend from genomic data.**
> Last paragraph, Introduction

Comment: *"The visualization in Figure 2C would be more informative if it included error bars to show the variance in confidence probabilities."*

Author reply: Thank you for your comment. Although we agree adding context to the distribution of the mean is important, the plot fo Figure 2C includes the distribution of all the points as a violin plot. Moreover, the plot shows, the distribution is not symmetric, which will turn the error bars deceiving.

Comment: *"The methods section should specify the exact number of training epochs and convergence criteria used. Some technical terms (e.g., "confidence probability" on page 5) should be defined more precisely."*

Author reply: We expanded the methodology to include the reviewer comment. We agree that including such information makes the paper easier to replicate. We introduce the following modifications to the main text:

> **The validation set is used to choose the parameters of the model (corresponding to the lowest validation loss during training) and the test set is not used during training. Figures in the SI explore different data splits. All the models in the main text were trained for 70-100 epochs.**
> First paragraph, Machine learning Implementation - Methodology

We also expand on the explanation of "confidence probability".

> TECSAS uses a softmax output layer, which means the output of each node can be related as a probability [5]. Each node represents a different subcompartment. The

model predicts the subcompartment for a locus by selecting the node with the highest probability. **Using these probabilities, we can extract the confidence of the model at predicting any specific subcompartment, i.e. the higher the prominent probability the more confident the model is at predicting, then, we refer as "confidence probability" as the output probability of the predicted subcompartment.**
Fourth paragraph, section 2.1

I. REVIEWER 3

Comment: *"Flexibility for Predicting Additional Subcompartments Recent evidence suggests that the human genome may contain more than the traditionally defined five subcompartments (e.g., Spracklin et al. 2019 https://doi.org/10.1038/s41594-022-00892-7, Zhang et al. 2024 bioRxiv http://dx.doi.org/10.48550/arXiv.2409.14425). It is essential that your method either be directly applicable or easily adaptable to scenarios with a greater number of subcompartments. If the model's current design inherently limits the number of subcompartments it can predict, this restriction will diminish its appeal to the broader chromatin biology community, where ongoing research increasingly points to a more complex compartmental landscape. Suggested improvements:*
- Please clarify whether the method can be readily extended to predict more than five subcompartments without extensive retraining or modification.
- Consider conducting experiments or analyses where you increase the number of subcompartment classes and report how the model's performance scales.
- Discuss the methodological adjustments required, if any, and provide guidance on how readers could adapt the approach for future, more fine-grained compartmental annotations."

Author reply: Thank you for your comment and suggestion. TECSAS can be easily adapted to predict different number of subcompartments. As our current results show, we can predict 5 (GM12878) or 4 (K562) subcompartments in section 2.1 - while in section 2.3, we show that the model can be expanded to predict 2 classes (XAD/nonXAD). In order to predict N subcompartments, one would only need to change the decoder layer with N output nodes. The model would need to be retrained as the 'new' decoder layer needs to be trained to predict the new set of subcompartments.
To facilitate this process, in Github, we provide an example highlighting how to train the decoder layer for 4 and 5 subcompartments. This tutorial allows to be trained N subcompartments from a different source.
Moreover, in section 2.3, we highlight the possibility of using A/B compartments along with Associated domains as a replacement for subcomparments - as shown in Figure 5, there could be 16

subcompartments based on all the combinations of compartments and XADS. As the reviewer points out, this show that one single representation of subcompartment may not be enough, therefore, a clasification like the one presented in Figure 5 can help to make subcompartment annotations interpretable based on openness (A/B) and their rate of association with nuclear bodies (XADs).

Comment: *"Mitigation of Train-Test Leakage and Proper Evaluation on Unseen Chromosomes The manuscript states that an 80/10/10 split (training/validation/testing) was used, but it is unclear how the authors avoided overlap between training and test regions, especially given that the model uses contiguous DNA segments as input. Overlapping sequences could lead to train-test leakage and artificially inflated performance. A best practice in genomic modeling tasks is to hold out entire chromosomes for testing, ensuring completely unseen data during evaluation. Suggested improvements: - Please describe the procedure in detail, explaining how you guaranteed that no overlap or partial overlap occurs between training and test regions. - Perform and report an additional experiment where you reserve entire chromosomes (or sufficiently large chromosome segments) as test data. - Provide metrics demonstrating the model's performance under these stricter conditions to validate that it generalizes well beyond the training sequences."*

Author reply: To address this comment, we proceed as the reviewer suggested. We trained the model in the two new conditions were the validation and test data correspond to different chromosomes. We trained both TECSAS and PYMB under two new conditions:

- Train set: Odd chromosomes loci
  Test set: every other loci in even chromosomes
  Validation set: Remaining loci in even chromosomes
  This condition correspond to roughly a 50/25/25 train, test and validation data split.
- Train set: 4-to-22 Chromosomes
  Test set: every other loci in chromosomes 1, 2 and, 3
  Validation set: Remaining loci in chromosomes 1, 2 and, 3.
  This condition correspond to roughly a 80/10/10 train, test and validation data split.

These two splits fully avoid data leakage between the training set and the evaluations sets. We introduced in the SI a new figure showcasing that regardless of the sticker data split, TECSAS outperforms PYMB.

Comment: *"Clarification of Test Data Usage in Figures and Comparisons It is not entirely clear which figures and results reflect performance on held-out test sets. The manuscript states*

that "Performance was assessed on held-out test sets," but it is ambiguous whether all reported metrics (e.g., in Figures 2 and 3) are derived from test data or a mixture of training, validation, and test sets. Additionally, the number of samples (genomic regions) underlying each figure is not fully disclosed. Finally, if different subsets of the genome were used for comparison to other methods (like PYMB), it is crucial to ensure that comparisons are made on identical, fully withheld test regions to avoid bias and ensure fair comparisons. Suggested improvements: - Clearly state which figures and results represent test-only evaluations and indicate the exact data subsets used. - Report the exact number of genomic regions analyzed for each figure. - Confirm that comparisons to PYMB and discrete TECSAS were conducted on the same genomic regions held out of training for your method. If this was not the case, please perform comparative analyses on the same withheld set. - If any reported results were obtained using the entire genome, please repeat those analyses using strictly test-only data and report these new outcomes. This will give readers confidence in the model's true generalizability."

Author reply: Thank you for your comment. All the reported metrics were computed for only test set - accuracy on train or validation sets are not reported. We introduced the following clarification on the text to address this:

> **The genome is segmented into train, test, and validation sets representing 80%, 10%, and 10% of all the loci, respectively (see Methods for more details). While validation set is used to select the models final set of parameters (parameters resulting on the lowest validation loss during training), all the reported metrics correponds to the model's performance at predicting the test set alone.**
> First paragraph, section 2.1

> **For the figures in the main text, the genome is split randomly forming training, validation, testing sets corresponding to 80%, 10% and 10% of all the loci, respectively. In the case of GM12878 (hg19 assembly), this corresponds to 41939 train loci, 5242 test loci and 5242 validation loci, while for all the other cell types (GRCh38 assembly), the data split is distributed as 45984 train loci, 5749 test loci and 5748 validation loci for XADs prediction at 50 kbp and 85652 train loci, 10706 test loci, 10707 validation loci for K562 subcompartments at 25 kbp.**
> **The validation set is used to choose the parameters of the model (corresponding to the lowest validation loss during training) and the test set is not used during training. Figures in the SI explore different data splits. All the models in the main text were trained for 70-100 epochs.**
> Second and third paragraph, Methods - Machine Learning Implementation

Comment: *"Availability and Quality of Code and Tutorials The manuscript references a GitHub repository, but a direct link was not found. The availability and quality of associated code, notebooks, and tutorials are essential for transparency, reproducibility, and community adoption of your methodology. Without access to this repository, it is impossible for reviewers and future readers to verify results, adapt the code, or reproduce the analyses. Suggested improvements: - Provide a functioning and stable URL to the GitHub repository.*
- Ensure that the code is accompanied by clear instructions, documentation, and example scripts that allow other researchers to replicate your results and apply the method to new data."

Author reply: Thank you for bringing this issue to our attention - our apologies for this oversight. We have created a new GitHub repository with the core code (main architecture of TECSAS) and a set of tutorials to load and train the model, making the model the scripts more accessible. We also provide the weights for the models, which are accessible through the GitHub. In the main text, we have included the following clarification:

> **TECSAS code is available in https://github.com/ed29rice/TECSAS.git. In the repository training examples and training procedures are included.**
> Last paragraph, section 4.2.1

Comment: *"Error Bars and Descriptive Statistics in Figures: - In Figure 3C, only the mean and maximum values are shown. Please include standard deviations or another measure of variability to offer a more complete statistical description. - If Figure 3A (and other figures) involves averaging over multiple instances, please also include error bars or confidence intervals to help readers interpret the stability of the reported metrics."*

Author reply: Thank you for you suggestion. We introduced error bars to figure 3C to better portray the data of the paper. Figure 3A correspond to single instances, therefore there are no error bars to report.

Comment: *"In Figure 4, the captions for panels A and C appear to be mixed up. Please correct and ensure that figure panels are described accurately."*

Author reply: Thank you for pointing this out. We changed the Figure caption to correctly refer each figure panel.

> **(A) Compartment annotations from TECSAS, PYMB and experimental Hi-C around the chr4:36-37Mbp segment. (B) Representative structure of chromosome 4 from simulations based on TECSAS and PYMB predictions, highlighting the positioning of the chr4:36-37Mbp segment. (C) Distribution of radial**

positioning of the chr4:36-37Mbp segment on the simulated ensemble based on TECSAS and PYMB annotations.
Caption, Figure 4.

Comment: *"Typos and Grammatical Errors: - Page 7, second paragraph from the end: "similar trend is observe between B1 and B2/3" → should be "observed." - Page 9, middle of paragraph 2: "ensembles based on the the predicted IMR-90 subcompartments" → remove the second "the." - Page 7, beginning: "PyMEGABASE (PYMB), another method that also only utilizes epigenomic data for predicting structural annotations" → Please rephrase this sentence for clarity and grammatical correctness. A thorough proofreading of the manuscript for English language usage, as well as attention to detail in figure captions and textual consistency, would substantially improve the readability and professionalism of the article."*

Author reply: We appreciate such as throughout read of our manuscript. We corrected those grammatical and typos. The manuscript now reads:

> **... similar trend is observed between B1 and B2/3.**
> Second paragraph, section 2.2

> **... ensembles based on the predicted IMR-90 subcompartments**
> Sixth paragraph, section 2.2

> **... The PYMB method also only utilizes epigenomic data for predicting structural annotations.**
> First paragraph, section 2.2

REVIEWER 4.

Comment: *"Training data and train/test splits: It is not immediately obvious from the text how the 80%, 10%, and 10% splits are generated. If random how do you ensure there is no data leakage between train and test? Is there a case where you can pre-train on one dataset and test on a different dataset to show the model generalises?"*

Author reply: Thank you for raising this point, we addressed this in a comment above. In summary, we introduced an SI figure training the model in the following conditions:

- Train set: Odd chromosomes loci
  Test set: every other loci in even chromosomes
  Validation set: Remaining loci in even chromosomes
  This condition correspond to roughly a 50/25/25 train, test and validation data split.

- Train set: 4-to-22 Chromosomes
  Test set: every other loci in chromosomes 1, 2 and, 3
  Validation set: Remaining loci in chromosomes 1, 2 and, 3.
  This condition correspond to roughly a 80/10/10 train, test and validation data split.

Regardless of the data split (avoiding any data leakage) our deep learning model outperforms PYMB.

Comment: *"This point about training and hold-out is valid for all datasets used in the analysis section of the paper. Please provide details how it is done for each results section where you train TECSAS and how you ensure appropriate train/test split?"*

Author reply: Thank you for you comment. We expanded the methodology and the main text to include more information about how the train, validation and test sets were selected. Here are the clarifications introduced:

> **The genome is segmented into train, test, and validation sets representing 80%, 10%, and 10% of all the loci, respectively (see Methods for more details). While validation set is used to select the models final set of parameters (parameters resulting on the lowest validation loss during training), all the reported metrics correponds to the model's performance at predicting the test set alone.**
> First paragraph, section 2.1

> **For the figures in the main text, the genome is split randomly forming training, validation, testing sets corresponding to 80%, 10% and 10% of all the loci, respectively. In the case of GM12878 (hg19 assembly), this corresponds to 41939 train loci, 5242 test loci and 5242 validation loci, while for all the other cell types (GRCh38 assembly), the data split is distributed as 45984 train loci, 5749 test loci and 5748 validation loci for XADs prediction at 50 kbp and 85652 train loci, 10706 test loci, 10707 validation loci for K562 subcompartments at 25 kbp.**
> **The validation set is used to choose the parameters of the model (corresponding to the lowest validation loss during training) and the test set is not used during training. Figures in the SI explore different data splits. All the models in the main text were trained for 70-100 epochs.**
> Second and third paragraph, Methods - Machine Learning Implementation

Comment: *"How many examples for each compartment there are? Are some classes unbalanced? This is again unclear from the text/supplementary."*

Author reply: Thank you for your question. We introduced an SI table describing the distribution of subcompartments for both GM12878 and K562 (Table S2). Although subcompartments are not perfectly balanced, they all have a similar order of magnitude number of samples. Interestingly, Figure 1 shows that more samples do not imply better prediction, as the subcompartment A1 is better predicted than A2.

Comment: *"Section 2.2: In this section you compare TECSAS to PyMEGABASE. From the text it seems there is another version of the TECSAS model for that task, so to that end you need to make it obvious from the paper how many versions of the model you've trained, etc. What are the experiments in Figure B? Maybe provide a table in the supplementary? Again what training data? Train-test splits, etc?"*

Author reply: We introduced a table describing the models we trained in the manuscript, describing how they were trained and implemented. We introduced the following to the main text:

> **In this paper, we trained the same architecture shown in Fig 1 for several use cases exemplified in the following table. Independent training, test, and validation sets were generated for models where it was trained and predicted in the same cell type.**
> Section 4.2

Comment: *"Figure 3: Figures A and B can benefit from a statistical test (+ multiple test correction). See comments above about fine-tuning task too: number of data points for each compartment, etc."*

Author reply: Thank you for pointing at this. In the case of Figure A, we are shown only 1 value per methodology (PYMB, TECSAS with discrete data, and TECSAS with continous signal). As for Figure 3B, we computed the non-parametic Mann Whitneyu p-value between PYMB and TECSAS accuracy distributions at each number of experiments, all of them were bellow 10^{-9}. Then, to address this comment, we introduced the following to the caption of Figure 3B:

> **... Prediction accuracy as a function of the number of input experiments for both PYMB and TECSAS, highlighting the consistent outperformance of TECSAS regardless of the number of features used (Mann Whitneyu p-value $< 10^{-9}$ between any PYMB and TECSAS distribution). ...**

Comment: *"No actual link to github: Availability is mentioned but no access to the code currently."*

Author reply: Thank you for pointing this oversight. We have created a new GitHub repository with the core code (main architecture of TECSAS) and a set of tutorials to load and train the model, making the model the scripts more accessible. We also provide the weights for the models, which are accessible through the GitHub. In the main text, we have included the following clarification:

> **TECSAS code is available in https://github.com/ed29rice/TECSAS.git. In the repository training examples and training procedures are included.**
> Last paragraph, section 4.2.1

Comment: *"The explanation of transformers can do with a bit of tightening. The first explanation of transformers perhaps can be rewritten to allow for readers without expert knowledge in the field and the explanation can be extended in Methods."*

Author reply: As the reviewer suggested, we have move the technical description of the transformer to the Methods section and instead introduced TECSAS architecture in a more accessible way. We have introduced the following to the main text:

> **As shown in Figure 1B, TECSAS architecture consists of several key components. First, *an input embedding* which transforms the epigenetic profile into a high-dimensional representation suitable for later interpretation. Second, *a positional fixed signal (positional embedding)* is added to the embedded signal to allow the model to understand the relative positioning between epigenetic signals. The resulting representation of the epigenetic signal is passed through *a transformer encoder* - this layer is in charge of interpreting the epigenetic profile and its internal relationships. Finally, the output from the encoder is decoded by *a linear layer+SoftMax layer* resulting in a probability distribution over the possible structural annotations - while predicting, the model assigns the most likely annotation based on the predicted probabilities.**
> Third paragraph, section 2.1

- **Input Embedding: The input epigenomic profile is first processed through a linear embedding layer, which transforms the data into a higher-dimensional representation suitable for the Transformer encoder.**
- **Positional Encoding: Positional encoding is added to the embedded input to incorporate information about the relative positions of the loci within the epigenomic profile.**

- **Transformer Encoder: The core of the model is a Transformer encoder with multiple attention heads. The encoder uses self-attention mechanisms to learn complex relationships and dependencies between different epigenomic features across the input loci. This allows the model to capture the context and long-range interactions that influence chromatin structure.**
- **Linear and SoftMax Output: The output of the Transformer encoder is passed through a linear layer, followed by a softmax activation function. The softmax layer outputs a probability distribution over the possible structural annotations, allowing the model to assign the most likely annotation to each locus.**

Section 4.2

We appreciate this suggestion - we agree this way the manuscript reads better and is more accessible.

Comment: *“Figures axes labels can be made more readable, use larger fonts, etc. Add axes labels to figure 3E. Please check all your figures to ensure they align with good figure standards.”*

Author reply: We added axes labels for Figure 3E, as well as reformat Figure 3 and Figure 4 for better readability.

---

[1] L. P. de Lima Camillo, R. Sehgal, J. Armstrong, A. T. Higgins-Chen, S. Horvath, and B. Wang, bioRxiv , 2024 (2024).

[2] K. Ying, J. Song, H. Cui, Y. Zhang, S. Li, X. Chen, H. Liu, A. Eames, D. L. McCartney, R. E. Marioni, *et al.*, bioRxiv , 2024 (2024).

[3] E. Nguyen, M. Poli, M. G. Durrant, B. Kang, D. Katrekar, D. B. Li, L. J. Bartie, A. W. Thomas, S. H. King, G. Brixi, J. Sullivan, M. Y. Ng, A. Lewis, A. Lou, S. Ermon, S. A. Baccus, T. Hernandez-Boussard, C. Ré, P. D. Hsu, and B. L. Hie, **386**, eado9336.

[4] G. Brixi, M. G. Durrant, J. Ku, M. Poli, G. Brockman, D. Chang, G. A. Gonzalez, S. H. King, D. B. Li, A. T. Merchant, M. Naghipourfar, E. Nguyen, C. Ricci-Tam, D. W. Romero, G. Sun, A. Taghibakshi, A. Vorontsov, B. Yang, M. Deng, L. Gorton, N. Nguyen, N. K. Wang, E. Adams, S. A. Baccus, S. Dillmann, S. Ermon, D. Guo, R. Ilango, K. Janik, A. X. Lu, R. Mehta, M. R. K. Mofrad, M. Y. Ng, J. Pannu, C. Ré, J. C. Schmok, J. S. John, J. Sullivan, K. Zhu, G. Zynda, D. Balsam, P. Collison, A. B. Costa,

T. Hernandez-Boussard, E. Ho, M.-Y. Liu, T. McGrath, K. Powell, D. P. Burke, H. Goodarzi, P. D. Hsu, and B. L. Hie, “Genome modeling and design across all domains of life with Evo 2,”.

[5] H. Ramsauer, B. Schäfl, J. Lehner, P. Seidl, M. Widrich, T. Adler, L. Gruber, M. Holzleitner, M. Pavlović, G. K. Sandve, V. Greiff, D. Kreil, M. Kopp, G. Klambauer, J. Brandstetter, and S. Hochreiter, “Hopfield Networks is All You Need,” (2021), arXiv:2008.02217 [cs, stat].
