# Supplementary material for: Epigenetics is all you need: A transformer to decode chromatin structural compartments from the epigenome
Source: PLoS Comput Biol. 2025 Dec 3;21(12):e1012326. doi: 10.1371/journal.pcbi.1012326 (PMC12685209; doi:10.1371/journal.pcbi.1012326)
Supplement: Attachment — Submitted filename: response_to_reviews_2.pdf [file pcbi.1012326.s018.pdf]

PLoS Comp Bio Editors and Reviewers,

We thank the reviewers for their additional feedback. Revisions in the manuscript are marked in red. Below we respond to each point and indicate changes to the main text, figures, and code/tutorials.

REVIEWER #1

Comment: *"I have read the revised version of the paper, but I am afraid to say that I am not satisfied with the responses. In fact, it would appear that the previous work from the authors did NOT perform the requested benchmarks, and hence these benchmarks should be included in this MS following the advice from this preprint (see https://www.biorxiv.org/content/10.1101/2024.09.16.613342v3) written by two of the most prominent and experienced bioinformaticians/statisticians. Indeed, as pointed out by Anders & Huber, the DL-application community is not using the correct benchmarks, a concern I deeply share. What is more, the authors claim at the start of section 2.2, that the previous Potts-based models were benchmarked against linear regression and random forests, citing reference [23], but I have inspected ref [23] and this paper does not even contain the words "linear regression" or "random forests", nor could I find a figure in that paper that provides a direct comparison to these other ML-methods. So, this is very disturbing. I also checked ref [24] and could also not find a comparison to penalized regression or RF. I think it is particularly important, when people are implementing DL methods to benchmark the DL method against a simpler ML-tool such as penalized regression or Random Forests. So, the authors are kindly requested to include these benchmarks in this work to serve as role model for future DL-based studies!"*

Author reply:

We sincerely thank the reviewer for their thorough feedback and highlighting the Ahlmann-Eltze et al. (2025) recently published work, which underscores the critical need for rigorous benchmarking in deep learning (DL) applications for biology, as emphasized by Anders & Huber. We apologize for the confusion in our previous response regarding the benchmarks in reference [23] (the PyMEGABASE paper - https://doi.org/10.1016/j.jmb.2023.168180). The comparisons to less complex machine learning methods, including Random Forest, were performed but presented in Supporting Information Figure S3, not the main text, and we regret that this was not clearly communicated.

We fully agree with the reviewer and the recently published call for robust baselines to demonstrate the added value of complex architectures like Transformers. To address this concern directly, we have conducted new benchmarking experiments comparing TECSAS to some ML base-

lines. The results (new Figure S11) show that these baselines achieve an average accuracy of $\simeq$ 0.65 across subcompartments on the GM12878 test set, while TECSAS reaches 0.78. These analyses are reproducible via the new notebook `benchmarks_simple_models.ipynb` in our GitHub repository (https://github.com/ed29rice/TECSAS).

In addition, it is worth noting that the study by Ahlmann-Eltze et al. focuses on generative prediction of transcriptomic changes following gene perturbations. In contrast, TECSAS performs a classification task—predicting chromatin subcompartment annotations (A1/A2/B1/B2/B3) from 1D epigenomic profiles. This task leverages correlations within observed epigenomic data, where the potential limitations of pre-training are less directly relevant to our classification task. Our primary baseline, PyMEGABASE (PYMB), a physics-inspired Potts model, was chosen because it outperforms simpler ML methods for this task (as shown in [23], Figure S3) and represents the state-of-the-art for epigenomic-based structural predictions. TECSAS has improved accuracy over PYMB (even when using less context/neighbors information - Figure 3A).

To further contextualize our approach and align with the call for more rigorous benchmarking, we have modified Section 2.2 of the manuscript to include this part:

> **To contextualize TECSAS's performance and align with discussions for rigorous Deep Learning benchmarking strategies in biology (e.g., Ahlmann-Eltze et al., 2025), we conducted benchmarks against different machine learning architectures (Figure S11).**

Comment: *"My 2nd major concern was also not addressed. I asked the authors to please provide a form of validation across different cell-types or using separate experiments generated on the same cell-line, but the authors failed to address this. It is simply unacceptable, in light of the above false statements, to claim that this was done in "previous works". The reader wants to know, if TECSAS is trained on cell-type "A" that it can predict subcompartments in cell-type "B". Or alternatively, if you train on cell-types "A" and "B" can you predict subcompartments in cell-type "C". Based on the table provided in the MS, the authors are clearly not performing the validations in the manner that I had advised."*

Author reply:

We sincerely thank Reviewers 1 and 4 for emphasizing the importance of cross-cell generalization, which has strengthened our manuscript by prompting clearer validation of TECSAS's transferability. We apologize for any lack of clarity in our previous response and appreciate the opportunity to address this concern directly.

As described in the manuscript (Section 2.3), the TECSAS encoder, trained on GM12878, was adapted by fine-tuning only the final linear layer to predict binary classifications (LAD/non-LAD,

NAD/non-NAD, SPAD/non-SPAD) for additional cell lines, including H1, HCT116, and IMR-90. These results, achieving accuracies of 0.78–0.85 (Figure 5A), demonstrate that the encoder's learned epigenomic features are transferable across cell types for predicting nuclear body associations. Furthermore, IMR-90 predictions were validated against 3D DNA-tracing data (Figure 5C), a cell line and data set fully independent of the training set.

To directly address the reviewers' request for cross-cell subcompartment prediction, we conducted a zero-shot experiment where the TECSAS model (encoder and decoder) trained on GM12878's 5-state subcompartment annotations was applied to predict A/B compartments in K562. The primary challenge, as noted, is the lack of experimentally derived 5-state subcompartment annotations for K562, as GM12878 is unique in having such high-resolution annotations from Hi-C data [Rao2014 - 10.1016/j.cell.2014.11.021]. Instead, we extracted A/B compartment annotations for K562 from eigenvector decomposition of Hi-C maps (ENCODE: ENCFF621AIY) using juicer_tools [Durand2016 - 10.1016/j.cels.2016.07.002]. TECSAS achieved an accuracy of 0.72 for A/B compartment predictions in K562, compared to 0.87–0.93 in GM12878 (Figure 2B), and outperformed PyMEGABASE (0.62) and simpler baselines (0.60–0.65) like Random Forests (Figure S11). These results are presented in the new Figure S9. This zero-shot experiment complements the manuscript's existing cross-cell results for XADs and aligns with TECSAS's goal of predicting compartments label from epigenomic data alone, reducing the dependence on Hi-C maps for that task. To align the manuscript with the response and incorporate the cross-cell generalization results, add the following paragraph to Section 2.3 (after the discussion of XAD predictions):

> **To further validate TECSAS's generalizability across cell types, we performed a zero-shot experiment where the complete model (encoder and decoder), trained on GM12878's 5-state subcompartment annotations, was applied to predict A/B compartments in the K562 cell line. We used A/B compartment annotations derived from PC1 of Hi-C eigenvector decomposition (ENCODE: ENCFF621AIY) via juicer_tools. TECSAS achieved an accuracy of 0.72 (Figure S9), compared to 0.87–0.93 in GM12878 (Figure 2B), and outperformed PyMEGABASE (0.68) and simpler baselines (0.60–0.65).**

<u>**Comment:**</u> *"My 3rd major concern was that there was a lack of understanding WHY TECSAS does better than the Potts Model. I argued that based on Fig.3DE, there is no evidence that the improvement comes from higher-order interaction terms, and indeed Fig.3D does NOT display a higher order interaction. The prediction of the locus in Fig.3D is clearly driven by the pairwise interaction of two marks H3K27me3 and H3K9me3, and whether their interaction is non-linear or linear does not address the issue that this pattern does not involve a 3rd type of histone*

mark or data-type. Whilst I agree with the authors that the attention-mechanism can in principle capture higher-order interactions, that does not mean that it actually does! Where is the example demonstrating this? After all, the author claim in the MS that their model is interpretable and that they can visualize higher-order interactions, but no figure is shown to demonstrate this."

Author reply: We thank the reviewer for this crucial and insightful question, which touches upon a key advantage of the Transformer architecture. We agree that the term "higher-order interaction" can be ambiguous, and we apologize for not defining it more precisely in the context of our model. While the self-attention mechanism in Transformers can theoretically capture such interactions by weighting all input features contextually across multiple heads and layers (Vaswani et al., 2017), we acknowledge that direct visualization is challenging and that Fig. 3D primarily highlights pairwise enrichments. The true strength of the Transformer, however, lies in how it models interactions dynamically and conditionally, which is fundamentally different from a static pairwise model like a Potts model. In a Potts model, the interaction between two features (e.g., H3K27me3 at exp i and H3K9me3 at exp j) is defined by a fixed coupling term J_{ij}. This term is learned from the data, but once learned, it is static. It captures the average pairwise correlation between these two features across the entire training set. However, to run the predictions, the node i is set to be the compartment label, and j is the different experiment. So, the contribution of each experiment j will determine what is the label i that minimizes the Potts energy function. So, this interaction is what we consider pairwise only. In the Transformer's self-attention mechanism, the "interaction" between the same two features is not fixed. Instead, the attention weight that feature j pays to feature i is calculated dynamically and is conditional on the entire epigenomic context. The influence of H3K9me3 at locus j on the representation of H3K27me3 at locus i is modulated by the presence and values of all other features in the neighborhood and determine the label of the locus i. This conditionality is the essence of what we mean by "higher-order." For instance, the model can learn a rule like: "Pay strong attention to the repressive mark H3K27me3 at L-7 IF AND ONLY IF there is also an absence of the active mark H3K36me3 at L-2 AND the presence of a high signal of a specific transcription factor at L+3." We consider this a non-linear interaction that a simple pairwise model cannot capture. It is not just an interaction between two marks, but an interaction between two marks that is switched on or off by a third feature, and the collection of these interactions will determine the label of the compartment of a particular locus. We acknowledge the significant challenge in visualizing such a high-dimensional, conditional dependency in a 2D plot. The attention maps in Figure 3D-E show the outcome of this complex calculation—the final weights—but they cannot fully unpack the conditional logic that produced them. This is an interpretability challenge in deep learning in general. To better reflect this in the manuscript and avoid ambiguity, we have revised the text in Section 2.2. Instead of focusing on the term "higher-order interactions," we now more precisely describe the model's ability to capture complex, conditional relationships across the epigenomic landscape. We have modified the

paragraph to read:

> **Unlike PyMEGABASE, which is based on a Potts model and captures fixed pairwise relationships, TECSAS utilizes a Transformer architecture with self-attention. One can consider that the attention layer mechanism can be related to the coupling matrix J_{ij} presented in the Potts model, although a direct comparison is not totally straightforward. The self-attention allows the model to learn complex, conditional relationships where the entire local epigenomic context dynamically modulates the interaction between any two epigenomic features. The importance the model assigns to a specific histone mark is not constant but can change depending on the presence or absence of other marks and transcription factors in the vicinity. While directly visualizing these high-dimensional dependencies is challenging, the attention maps summarize the most influential connections the model uses to inform its predictions.**

Comment: *"Related to the previous point, It is also clear that a significant improvement derives mainly from considering larger neighborhoods (Fig.3E), but whether a simpler ML model using larger neighborhoods results in similar prediction accuracies is a key question not explored by the authors. Therefore, the reader has no clear understanding what is driving the improvement. This issue is perfectly addressable, and that the authors are refusing to address it raises concerns, thus compounding earlier concerns about the lack of appropriate benchmarking."*

Author reply: We thank the reviewer for highlighting this important point and apologize for not addressing it more explicitly in prior revisions. We agree that disentangling the contributions of larger neighborhoods from the Transformer architecture is essential to understanding TECSAS's improvements over baselines. To address this, we performed additional training and predictions extending PyMEGABASE (PYMB) to use varying neighborhood sizes (Figure S10). Different ML methods are presented in Figure S11 (best run).

Comment: *"A fifth concern, which I did not mention in my earlier review, is that there is also a lack of a figure that clearly compares methods numerically in terms of some objective evaluation metric. For instance, Fig.4A shows one cherry-picked example where TECSAS performs better than PYMB, but where is the panel comparing validation correlation coefficients with Hi-C data across all cell-lines? You can't just cherry-pick one region where TECSAS performs better if we could find other regions where PYMB does better."*

Author reply: We thank the reviewer for raising this valid concern. To address this, we conducted a genome-wide locus-by-locus comparison of TECSAS and PYMB against experimental

Hi-C-derived annotations (eigenvector PC1) for IMR-90 and GM12878 (Figure S12). We also identified a region close to the centromere where PYMB performs better than TECSAS (Figure S13).

REVIEWER #3

Comment: *"I appreciate the authors' considerable efforts to refine both the manuscript and its accompanying codebase. The manuscript is now substantially stronger, and my earlier comments have been satisfactorily addressed. I was able to access and run the code without difficulty; however, the online description of the TECSAS instrument remains rather concise. Expanding the tutorials—especially by pinpointing the key steps required for data and intermediary quality control—would greatly aid prospective users in adopting TECSAS with confidence. At present, the codebase still feels somewhat pared-down, and additional explanatory material would enhance its accessibility."*

Author reply:

We sincerely thank the reviewer for their positive feedback and constructive suggestions regarding the enhancement of TECSAS documentation and tutorials. We are pleased that the revisions have strengthened the manuscript and that the codebase is now accessible and functional. We fully agree that expanding the tutorials would greatly benefit user adoption and have updated the GitHub repository accordingly. Specifically, we have added more detailed comments and annotations to the existing notebooks, along with a new section in the README file outlining key steps for data preparation, model training, and basic troubleshooting. The suggestion for enhanced quality control measures is valuable and is incorporated in the initial tutorials, including checks for input data consistency (e.g., signal normalization and locus resolution matching). However, we would like to clarify an important aspect of our data handling approach: TECSAS is primarily designed to work with data from the ENCODE database, which has already undergone rigorous processing and curation standards. This ensures users start with validated, high-quality datasets. Users also have the option to incorporate their own datasets, and TECSAS includes basic checks to verify if the data are in the correct format. That said, these checks do not extend to assessing the overall quality of the input experiments, as this falls outside the scope of the platform at this point, but could be included in future versions of the platform.

REVIEWER #4
Major

Comment: *"1. Dataset: I understand that the training, test, and validation are split based on chromosomes but if using sequences it's good practice to think about the similarity of those sequences to prevent data leakage. Can we quantify this to ensure there isn't any data leakage?"*

Author reply:

Thank you for your comment and for highlighting the importance of preventing data leakage in genomic models. We would like to clarify that our model does not utilize DNA sequence data as input. Instead, TECSAS relies exclusively on epigenetic features(e.g., signal intensities from histone modification ChIP-seq, transcription factor ChIP-seq, and RNA-seq), processed locus by locus at a fixed resolution (25 kbp or 50 kbp). These signals represent aggregated experimental measurements of chromatin states (e.g., histone modifications and transcription factor binding) rather than raw nucleotide sequences. Given that the predictions are performed locus-by-locus and long segments of repetitive chromatin types A or B are expected, we compared the difference between the training and test set performance, which could be a proxy for data leakage if the performance does not reach similar accuracy. This is not the case here, where TECSAS performed well in the test set (Figure S14).

Comment: *"2. Table 1 is very helpful in understanding what you have done but I am still unclear on whether the model generalises. What is the baseline performance of your model? What happens if you use the model trained on GM12878 (both encoder and decoder) and predict K562? That would indicate what generalisable features are learned and to what extent the model can be used when not enough data specific to a cell type is available."*

We sincerely thank Reviewers 1 and 4 for emphasizing the importance of cross-cell generalization, which has strengthened our manuscript by prompting clearer validation of TECSAS's transferability. We apologize for any lack of clarity in our previous response and appreciate the opportunity to address this concern directly.

As described in the manuscript (Section 2.3), the TECSAS encoder, trained on GM12878, was adapted by fine-tuning only the final linear layer to predict binary classifications (LAD/non-LAD, NAD/non-NAD, SPAD/non-SPAD) for additional cell lines, including H1, HCT116, and IMR-90. These results, achieving accuracies of 0.78–0.85 (Figure 5A), demonstrate that the encoder's learned epigenomic features are transferable across cell types for predicting nuclear body associations. Furthermore, IMR-90 predictions were validated against 3D DNA-tracing data (Figure 5C), a cell line and data set fully independent of the training set.

To directly address the reviewers' request for cross-cell subcompartment prediction, we con-

ducted a zero-shot experiment where the TECSAS model (encoder and decoder) trained on GM12878's 5-state subcompartment annotations was applied to predict A/B compartments in K562. The primary challenge, as noted, is the lack of experimentally derived 5-state subcompartment annotations for K562, as GM12878 is unique in having such high-resolution annotations from Hi-C data [Rao2014 - 10.1016/j.cell.2014.11.021]. Instead, we extracted A/B compartment annotations for K562 from eigenvector decomposition of Hi-C maps (ENCODE: ENCFF621AIY) using juicer_tools [Durand2016 - 10.1016/j.cels.2016.07.002]. TECSAS achieved an accuracy of 0.72 for A/B compartment predictions in K562, compared to 0.87–0.93 in GM12878 (Figure 2B), and outperformed PyMEGABASE (0.62) and simpler baselines (0.60–0.65) like Random Forests (Figure S9). These results are presented in the new Figure S10 and Table S4.

This zero-shot experiment complements the manuscript's existing cross-cell results for XADs and aligns with TECSAS's goal of predicting compartments label from epigenomic data alone, reducing the dependence on Hi-C maps for that task. To align the manuscript with the response and incorporate the cross-cell generalization results, add the following paragraph to Section 2.3 (after the discussion of XAD predictions):

> **To further validate TECSAS's generalizability across cell types, we performed a zero-shot experiment where the complete model (encoder and decoder), trained on GM12878's 5-state subcompartment annotations, was applied to predict A/B compartments in the K562 cell line. We used A/B compartment annotations derived from PC1 of Hi-C eigenvector decomposition (ENCODE: ENCFF621AIY) via juicer_tools. TECSAS achieved an accuracy of 0.72 (Figure S10, Table S4), compared to 0.87–0.93 in GM12878 (Figure 2B), and outperformed PyMEGABASE (0.68) and simpler baselines (0.60–0.65).**

Comment: *"3. Thanks for adding the link to the GitHub but if this is to be widely used there are things needed like guide to installation/requirements file. Tutorial notebooks are lacking comments, so more documentation and details."*

Author reply: We appreciate the reviewer's feedback on the GitHub repository (https://github.com/ed29rice/TE and agree that enhanced documentation is essential for broader adoption. To address this, we have expanded the repository with a comprehensive installation guide, including a requirements file for easy setup. Additionally, we have added detailed comments throughout the tutorial notebooks, along with step-by-step explanations of the TECSAS pipeline, including data preparation, model training, and testing protocols. These updates include example workflows and troubleshooting tips to make the tool more accessible and reproducible.

Minor

Comment: *"Some of the figures and table references are not working and appear as ? in the text"*

Author reply: Thank you for pointing out the reference issues in the manuscript. These are artifacts from the LaTeX compilation process during the manuscript formatting into the submission platform. We have resolved the compilation errors and will ensure that the final submission includes a PDF with all figures, tables, references, and citations correctly resolved and displayed.

Comment: *"Figure 2C is a bit difficult to read. I get that it's showing that you are fairly confident with some lower confidence for some compartments but perhaps the visualisation can be improved to make it easier to understand."*

Author reply: We thank the reviewer for their observation on Figure 2C. We have reviewed the visualization and believe it now conveys the distribution of confidence probabilities across subcompartments, with the violin plots highlighting both the central tendencies and variability. To enhance clarity, we have increased the font sizes for labels.
